# Fatal Meningitis in Patient with X-Linked Chronic Granulomatous Disease Caused by Virulent *Granulibacter bethesdensis*

**DOI:** 10.3201/eid2505.181505

**Published:** 2019-05

**Authors:** Mafalda Rebelo, Li Ding, Ana Isabel Cordeiro, Conceição Neves, Maria João Simões, Adrian M. Zelazny, Steven M. Holland, João Farela Neves

**Affiliations:** Hospital Dona Estefânia—Centro Hospitalar de Lisboa Central, Entidade Publica Empresarial, Lisbon, Portugal (M. Rebelo, A.I. Cordeiro, C. Neves, J.F. Neves);; National Institute of Allergy and Infectious Diseases of the National Institutes of Health, Bethesda, Maryland, USA (L. Ding, A.M. Zelazny, S.M. Holland);; National Institute of Health Dr. Ricardo Jorge, Lisbon (M.J. Simões);; Chronic Diseases Research Center, NOVA Medical School, Lisbon (J.F. Neves)

**Keywords:** Chronic granulomatous disease, Granulibacter bethesdensis, meningitis/encephalitis, virulent strain, X-linked defect, bacteria, immunodeficiency

## Abstract

*Granulibacter bethesdensis* is a pathogen reported to cause recurrent lymphadenitis exclusively in persons with chronic granulomatous disease. We report a case of fatal meningitis caused by a highly virulent *G. bethesdensis* strain in an adolescent in Europe who had chronic granulomatous disease.

Chronic granulomatous disease (CGD) is a primary immunodeficiency characterized by a deficient nicotinamide adenine dinucleotide phosphate oxidative burst that impairs phagocyte superoxide formation and killing of certain pathogens. Mutations can occur in any of the 5 subunits of nicotinamide adenine dinucleotide phosphate oxidase. Most cases are inherited as X-linked defects (gp91*^phox^*), but they also can occur in an autosomal recessive manner (*1*). Increased susceptibility develops to recurrent infections of the skin, lymph nodes, lungs, and other organs (*2*), mostly caused by bacteria and fungi, including *Staphylococcus aureus*, *Serratia marcescens*, *Burkholderia cepacia*, *Salmonella* spp., *Nocardia* spp., and *Aspergillus* spp. (*2*). Emerging organisms, such as *Granulibacter bethesdensis* and other methylotrophs, occur almost exclusively in CGD patients (*3*,*4*).

*G. bethesdensis* was first described in 2006, when it was isolated in a CGD patient with lymphadenitis (*4*). It is a gram-negative, aerobic, oxidase-negative, catalase-positive, nonmotile coccobacillus to rod-shaped bacterium belonging to the *Acetobacteraceae* family (*5*,*6*). *G. bethesdensis* was the first of these *Acetobacteraceae* family bacteria with proven pathogenicity in humans, causing invasive disease in CGD patients and mice (*4*). It has been mostly linked to indolent nonfatal lymphadenitis and deep neck infections in patients in North America. The infection can recur over several years by reactivation of the same strain or reinfection with different strains (*3*,*7*–*9*). The first fatal infection was reported in a 10-year-old boy from Spain, who died of fulminant sepsis (*10*). In vitro, *G. bethesdensis* shows extensive resistance to various antimicrobial drugs, although its slow growth makes susceptibility testing difficult. Ceftriaxone, aminoglycosides, doxycycline, and trimethoprim/sulfamethoxazole showed activity in vitro (*7*).

We report a case of *G. bethesdensis* meningitis in a patient with X-linked CGD. We also report animal data comparing this *G. bethesdensis* strain with the strain recovered from recurrent lymphadenitis in a US CGD patient.

## The Study

**Figure 1:**
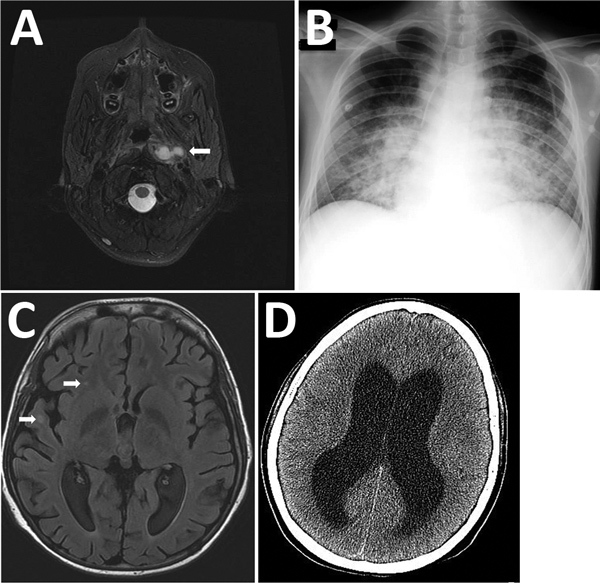
Most relevant imaging results from a 16-year-old boy with X-linked chronic granulomatous disease who died of meningitis caused by a virulent *Granulibacter bethesdensis* strain. A) Computed tomography image showing deep cervical abscess (arrow), July 2012. B) Radiograph showing pneumonia with pleural effusion, October 2012. C) Cranial magnetic resonance image showing multiple intraparenchymal brain abscesses (arrows), December 2012. D) The patient died of obstructive hydrocephalus (shown) and multiorgan failure, April 2013.

The patient was a 16-year-old boy whose X-linked CGD (CYBB exon 13 deletion) was diagnosed when he was 2 years old. His disease had been well-controlled with cotrimoxazole, itraconazole, and interferon-γ. In September 2014, he was hospitalized with a deep cervical abscess (Figure 1, panel A) and received a 5-week course of intravenous ciprofloxacin, doxycycline, and ceftriaxone that resulted in complete clinical and radiologic resolution, followed by 6 weeks of oral amoxicillin/clavulanic acid, doxycycline, and ciprofloxacin along with his usual prophylaxis. No pathogen was identified despite blood cultures, bronchoalveolar lavage, and lymph node biopsy cultures and broad-range bacterial PCR.

After this regimen was completed, the boy was readmitted for 8 weeks with pneumonia with pleural effusion (Figure 1, panel B). Full 16S rRNA gene sequencing (≈1,500 bp) identified *Cupriavidus* spp. in pleural fluid. He received meropenem, amikacin, ciprofloxacin, teicoplanin, doxycycline, and voriconazole, and his condition improved. However, 2 weeks later, fever returned, along with splenomegaly, hemodynamic instability, pancytopenia, hypofibrinogenemia, hyperferritinemia, and elevated soluble CD25. He received intravenous immunoglobulin and dexamethasone for this inflammatory condition and fully recovered. Neck and lung computed tomography images and positron emission tomography performed 1 month later showed no signs of active infection.

Nevertheless, a few days later, the patient sought care for altered mental status, hallucinations, aggressiveness, and respiratory instability requiring admission to the pediatric intensive care unit. He had extensive bilateral pneumonia and multiple intraparenchymal brain abscesses (Figure 1, panel C). Meropenem, ciprofloxacin, amikacin, doxycycline, teicoplanin, and voriconazole were started; results of cerebrospinal fluid (CSF) and lung biopsy samples were unremarkable. Teicoplanin was switched to linezolid and voriconazole to caspofungin and liposomal amphotericin B because of toxicity concerns. Four weeks later, he was discharged from the intensive care unit. One month later, fever, vomiting, and focal neurologic deficits developed. CSF showed pleocytosis and hypoglycorrhachia with elevated protein levels. Cerebral imaging confirmed leptomeningitis. Isoniazid, clarithromycin, and rifampin were added to his treatment regimen, but his neurologic status continued to deteriorate. Obstructive hydrocephalus (Figure 1, panel D) and multiorgan failure developed, and he died 3 months later.

**Figure 2:**
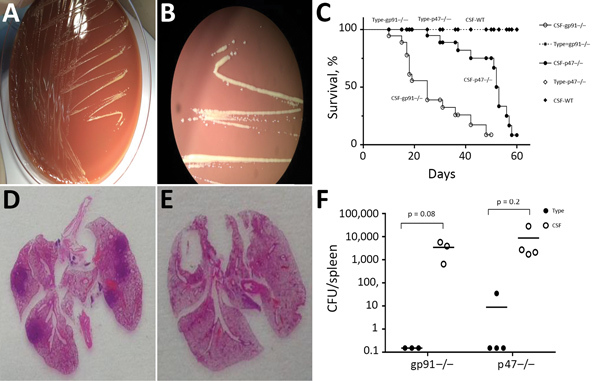
*Granulibacter bethesdensis* colonies and pathology results after inoculation on mouse models. A, B) CSF on chocolate agar for 4 days showed slowly growing brown-yellow, shiny colonies 2–4 mm in diameter. Full 16S RNA sequencing (≈1,500 bp) led to identification of *G. bethesdensis* (with 99.7% match). C) Survival of gp91/p47 KO mice after inoculation of different *G. bethesdensis* strains. D) Pathology images of gp91 KO mouse lung after CSF strain infection. E) Pathology images of gp91 KO mouse lungs after type strain infection. F) Quantification of *G. bethesdensis* strains in spleens of gp91/p47 KO mice after inoculation. CSF, cerebrospinal fluid; type, National Institutes of Health type strain; WT, wild type.

CSF cultures yielded yellow-brown, shiny, small colonies (2–4 mm) on chocolate agar after 4 day’s incubation. (Figure 2, panels A, B). Full 16S RNA sequencing (≈1,500 bp) showed 99.7% match to the type strain of *G. bethesdensis* CGDNIH1T (ATCC BAA-1260T, DSM 17861T) from North America and 100% match to a previously reported *G. bethesdensis* strain from Spain. Nonstandardized susceptibility test using Etest, performed in Mueller Hinton agar supplemented with 5% sheep blood agar with an overnight air incubation at 37°C, showed resistance to doxycycline (MIC 24 mg/L) and ceftriaxone (MIC >32 mg/L).

We used mouse models of CGD to determine whether differences existed in immune response, pathogenicity, or severity of disease between the European (CSF strain) and the US (type strain) strains. We intraperitoneally infected gp91*^phox−/−^* mice with 10^7^ CFU of *G. bethesdensis* type strain and p47*^phox−/−^* mice intraperitoneally with 10^7^ CFU of CSF strain and monitored moribundity during infection. We euthanized gp91*^phox−/−^* mice 4 weeks and p47*^phox−/−^* mice 8 weeks after infection and collected brain, spleen, lung, lymph nodes, and blood for culture, bacterial enumeration, and histopathologic examination. Plasma cytokines were assayed.

Although we observed differences between gp91*^phox−/−^* and p47*^phox−/−^* mice, both CGD mice models showed high rates of death when infected with CSF strain. No deaths occurred in mice infected with type strain, nor did CSF strain cause disease in wild-type mice (Figure 2, panel C). CSF strain–infected mice showed more severe pathologic organ changes than did type strain–infected mice (Figure 2, panels D, E; Appendix Figure 1). We performed quantitative cultures to assess bacterial load in the spleens of inoculated mice. CSF strain CFUs were 100–1,000 times higher than those of type strain 4 and 8 weeks after infection (Table; Figure 2, panel F). In addition, infection with CSF strain yielded 100–1,000 times higher CFUs in spleens of CGD mice than in wild-type mice 4 and 8 weeks after infection. (Appendix Figure 2). CSF strain showed faster growth on solid and in liquid media and a higher optimal growth temperature (37°C) than previously described North America lymph node isolates (Appendix Figure 3). CGD mice infected with CSF strain showed higher plasma interleukin-1β, tumor necrosis factor–α, and interleukin-6 than those infected with type strain 4 and 8 weeks after infection, which correlated with differences in tissue bacterial load. Cytokine levels did not increase in wild-type mice infected with CSF strain (Appendix Figure 4).

**Table:** Bacterial culture from blood and brain samples of GP91 KO mouse infected with *Granulibacter bethesdensis* after 4 weeks*

Mouse	Strain	Sample	
		Blood	Brain
gp91^−/−^, n = 10	Type, USA	0	0
gp91^−/−^, n = 10	**CSF, Portugal**	**5**	**2**
p47^−/−^, n = 5	Type	0	0
p47^−/−^, n = 5	CSF	0	0
WT, n = 9	CSF, Portugal	0	ND

*Bold indicates the results of the inoculation of CSF strain in the gp91 KO mouse. CSF, cerebrospinal fluid; ND, not done; WT, wild type.

## Conclusions

*G. bethesdensis* is an emerging pathogen shown to cause infection exclusively in CGD patients and has a spectrum of disease severity ranging from chronic and recurrent infections to fulminant sepsis, central nervous system infection, and death (*3*,*7*,*10*). Until recently, all reported North America cases were nonfatal chronic infections; 1 case from Europe (Spain) was fatal. Recently, Mayer et al. reported a X-linked CGD patient in the United States who died of fulminant infection with an organism with 100% identity to 500 bp of *G. bethesdensis* 16S (*11*). Unfortunately, that *G. bethesdensis* isolate was not available for analysis and comparison with other *G. bethesdensis* strains. The previous strain from Europe was highly resistant to antimicrobial agents, including colistin, most β-lactams, and quinolones (*10*).

We found that a CSF *G. bethesdensis* strain, showing an identical 16S sequence to a previously described fulminant strain from Europe, was more virulent and lethal in a mouse model than the *G. bethesdensis* US type strain and more virulent in gp91*^phox−/−^* than in p47 *^phox−/−^* mice. A fatal case of *G. bethesdensis* infection in the United States suggests that heterogeneity might exist among North America *G. bethesdensis* strains. Bacterial genome sequencing may identify discrete virulence factors. *G. bethesdensis* must be included as a cause of fatal disseminated infection in CGD.

Appendix Additional images of mice experimentally infected with virulent *Granulibacter bethesdensis* strain.
