# Supplementary material for: Fatal Meningitis in Patient with X-Linked Chronic Granulomatous Disease Caused by Virulent Granulibacter bethesdensis
Source: Emerg Infect Dis. 2019 May;25(5):976–9. doi: 10.3201/eid2505.181505 (PMC6478198; doi:10.3201/eid2505.181505)
Supplement: Appendix — Additional images of mice experimentally infected with virulent Granulibacter bethesdensis strain. [file 18-1505-Techapp-s1.pdf]

Article DOI: https://doi.org/10.3201/eid2505.181505

Fatal Meningitis in Patient with X-Linked Chronic Granulomatous Disease Caused by Virulent *Granulibacter bethesdensis*

Appendix

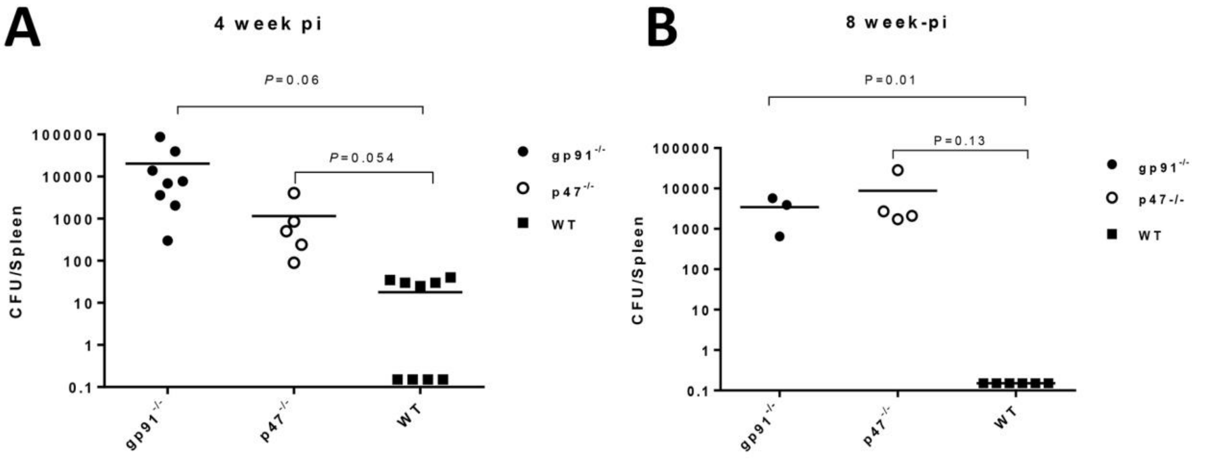

Appendix Figure 1. Histology images. A–F: Lung of gp91–/– mice infected with *Granulibacter bethesdansis* cerebrospinal (CSF) strain. (A) Lung: low magnification showing the multifocal areas of pneumonia. (B) Airway: bronchioles with neutrophils in the lumen, also lymphocytes around the airway and vessel. (C) Brown & Hopps staining bacteria (black arrow) in lung. (D) Lung: low magnification showing the diffuse nature of the pneumonia. (E) Pleura: inflammation extended to the pleura. (F) Degeneration: degenerative neutrophils and necrotic cell debris. (G) Brain of gp91$^{-/-}$ mouse infected with CSF strain: meningeal thickening and histiocyte infiltration-histiocytic meningitis. (H) Brain lesions of gp91$^{-/-}$ mouse infected with CSF strain: cell died. (I) Brain of gp91$^{-/-}$ mouse infected with type strain.

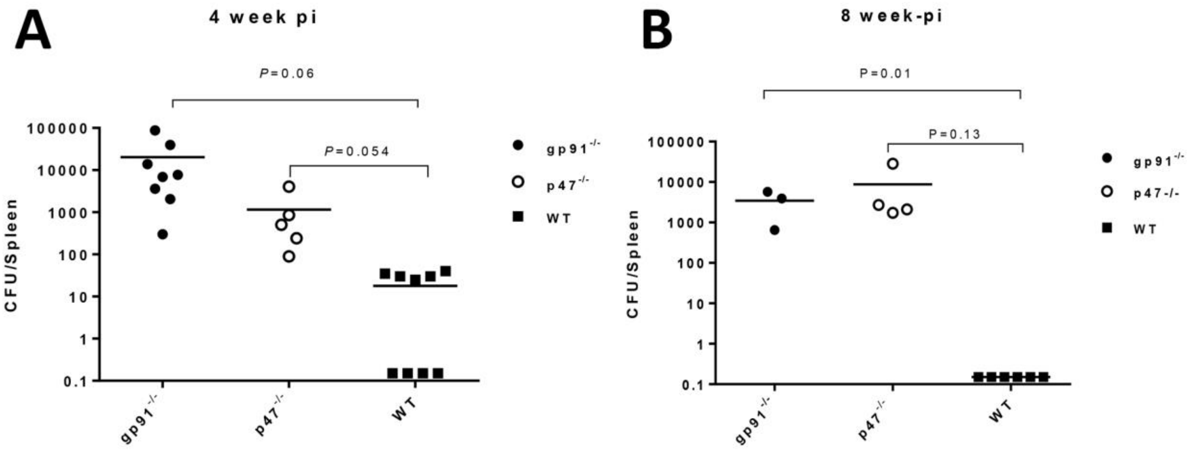

Appendix Figure 2. *Granulibacter bethesdensis* cerebrospinal fluid strain recovery from mice spleen homogenates 4 weeks and 8 weeks after infection.

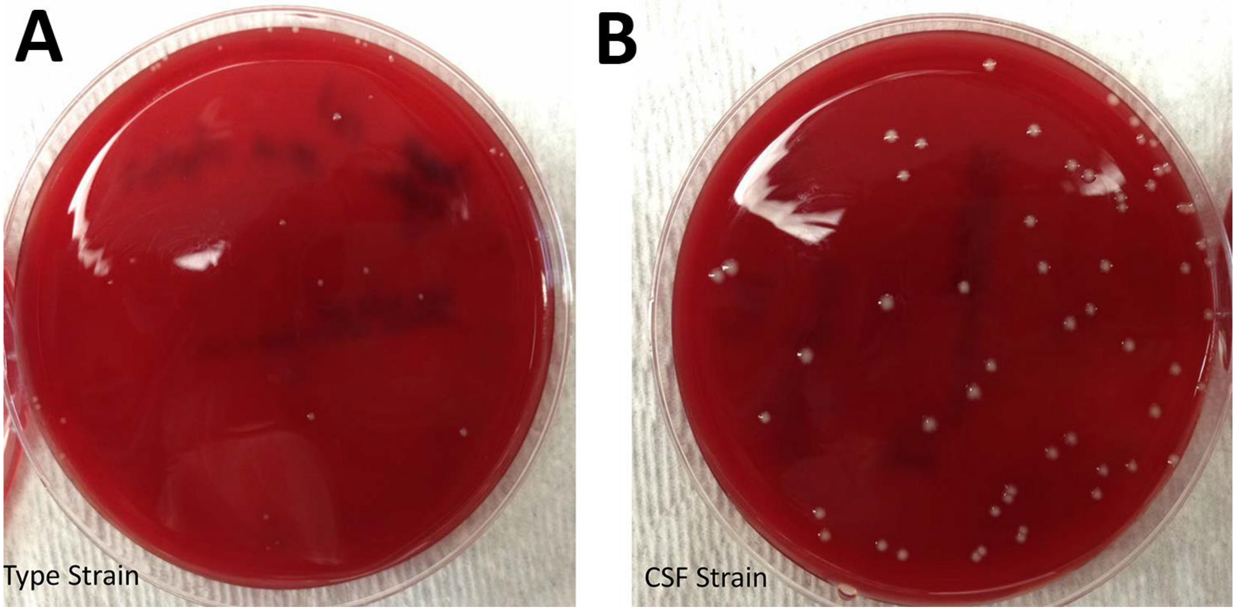

Appendix Figure 3. Growth comparison between type strain colonies (left) and cerebrospinal fluid strain colonies (right).

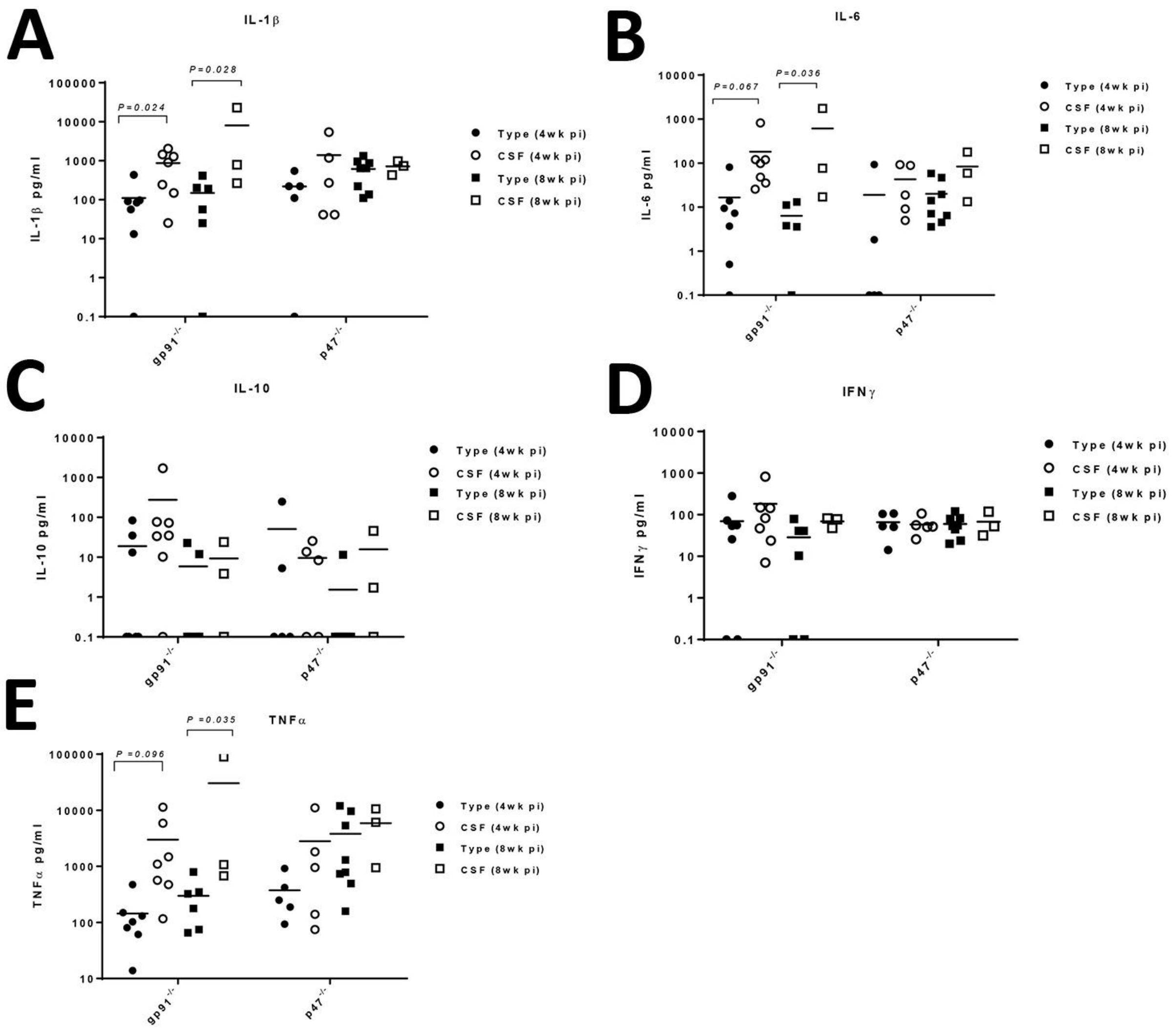

Appendix Figure 4. Cytokine production in *Granulibacter bethesdensis* (type & cerebrospinal fluid) infected mice plasma.
